# Evaluation of the therapeutic effects of rehabilitation therapy on patients with amyotrophic lateral sclerosis—a meta-analysis

**DOI:** 10.3389/fneur.2024.1389146

**Published:** 2024-05-03

**Authors:** Jianghua Cheng, Xiaomin Niu, Hui Li, Qiuwei Yang, Ketao Du

**Affiliations:** Department of Rehabilitation, South China Hospital, Medical School, Shenzhen University, Shenzhen, China

**Keywords:** amyotrophic lateral sclerosis, ALS, neurodegenerative disease, muscle atrophy, rehabilitation therapy

## Abstract

**Objective:**

To investigate the effect of rehabilitation therapy on the global function, respiratory function, and quality of life in patients with amyotrophic lateral sclerosis (ALS).

**Methods:**

PubMed, Web of Science, and The National Library of Medicine (NLM) were systematically searched and the search period was between the date of database establishment and December 31, 2023. The outcome measures finally analyzed included the ALS functional rating scale/revised (ALSFRS/ALSFRS-R), forced vital capacity percentage predicted (FVC%), fatigue severity scale (FSS), and maximal expiratory pressure (MEP).

**Results:**

A total of 13 randomized controlled trials (RCTs) were included, and 5 outcome measures were pooled and analyzed. A total of 657 patients with ALS were enrolled, with 299 in the experimental group (rehabilitation therapy, such as resistance training, endurance training, aerobic training, respiratory muscle training, and standard rehabilitation therapy) and 358 in the control group (conventional interventions, such as simple joint movements or daily stretching). The ALSFRS scores were better in the experimental group than in the control group at 0–4 months (MD = 3.36, 95% CI: 0.82, 5.91, *Z* = 2.59, *p* = 0.009) and at 5–8 months (MD = 5.00, 95% CI: −2.42, 7.58, *Z* = 3.80, *p* < 0.001). Moreover, the ALSFRS-R scores of the experimental group was better than that of the control group at 5–8 months (MD = 2.83, 95% CI: 1.21, 4.45, *Z* = 3.42, *p* < 0.001) and 9–12 months (MD = 1.87, 95% CI: −0.37, 4.11, *Z* = 1.63, *p* = 0.10). It was also found that the MEP value of the experimental group was significantly better than that of the control group after intervention (MD = 18.49, 95% CI: 1.47, 35.50, *Z* = 2.13, *p* = 0.03). However, there were no significant differences in FVC% value and FSS scores at 0–5 months and 6–12 months between the two groups.

**Conclusion:**

Rehabilitation therapy is helpful in improving the short-, medium-, and long-term global function score of patients with ALS, with positive effects on respiratory function.

## 1 Introduction

Amyotrophic lateral sclerosis (ALS) is a fatal neurodegenerative disease selectively involving the upper and lower motor neurons and tracts, with clinical manifestations of progressive muscle atrophy, bulbar paralysis and pyramidal sign (1). The main symptoms of ALS include myasthenia and spasms, which often lead to a decreased functional capacity in these patients. Studies have reported that the motor, speech, swallowing, and respiratory functions of patients with ALS are impaired to various degrees, with extremely poor quality of life (QOL) and prognosis (2). Respiratory insufficiency induced by musculoskeletal dysfunction is the leading cause of death in patients with ALS (3).

The goals of ALS treatment usually are to enhance the body function, improve the sense and feel, and reduce the incidence of adverse events to comprehensively improve the QOL of these patients. Rehabilitation therapy, pathological therapy and various supportive treatments are commonly used in the clinical management of patients with ALS. For example, there are various types of rehabilitation therapy, and physical, occupational, and speech therapies as well as other adjuvant therapy such as music therapy and art therapy, are the basic treatment methods among others (4). Physical therapy is subdivided into two categories, i.e., exercise therapy and physical therapy. Exercise therapy includes joint function training, muscle strength training, respiration training, aerobic exercise and ambulation training, and physical therapy commonly involves the use of artificial physical factors such as electricity, light, sound, magnetism, temperature and mechanical force (5).

Recent studies have suggested that rehabilitation therapy, including endurance training, aerobic training and respiration training, can improve muscle strength, slow down the degeneration of motor neurons, reduce the stress on fast muscle fibers, enhance muscle endurance, and improve cardiopulmonary function in patients with ALS (6, 7). At the same time, Garber et al. (8) proposed that physical exercise, one of the most common types of rehabilitation therapy for ALS, effectively improves the muscle strength and cardiovascular function and the QOL of these patients. A meta-analysis published in 2021 showed that therapeutic physical activities help slow the progression of ALS and improve the performance of the muscle tissue in the daily lives of these patients (9). However, another study has found that aerobic, resistance and combined exercise training showed no positive results in functional ability, respiratory function, fatigue, pain, quality of life, upper-body strength, lower-body strength, or Vo2peak (10). This difference may be related to the significant heterogeneity in the therapeutic effects of different studies on ALS due to the diversity of exercise programs used. The conflicting findings suggest that further investigations are needed to evaluate the impact of rehabilitation therapy on patients with ALS.

Therefore, publications about the effects of rehabilitation therapy on the global function, respiratory function, QOL and other parameters of ALS were collected and reported through systematic reviews and meta-analyses to summarize these evaluation indicators. On this basis, the effects of rehabilitation therapy on ALS were evaluated to provide references and suggestions for the clinical rehabilitation treatment of ALS.

## 2 Materials and methods

### 2.1 Search strategy

Three databases, including PubMed, Web of Science, and the National Library of Medicine (NLM), were systematically searched according to the Preferred Reporting Items for Systematic Reviews and Meta-Analyses (PRISMA) statement, and the search period was between the date of database establishment and December 31, 2023. This study has been prospectively registered in the PROSPERO (CRD42020209359). A combination of subject terms and free terms were used for literature retrieval, and the keywords included “amyotrophic lateral sclerosis or motor neuron disease” AND “rehabilitation or rehabilitation medicine therapy or respiratory muscle training or muscle strength training or aerobic exercise” AND “effect or clinic effect.”

### 2.2 Inclusion and exclusion criteria

Inclusion criteria included (1) study types: randomized controlled trials (RCTs); (2) study subjects: patients diagnosed with ALS who met one of the 4 commonly used diagnostic criteria: the EI Escorial criteria (1994) (11), the revised EL Escorial criteria (2000) (12), the Awaji (2008) (13), and the Gold Coast criteria (2020) (14). Patients definitely and probably diagnosed with ALS were included in this meta-analysis; (3) rehabilitation therapy such as including resistance training, endurance training, aerobic training, respiratory muscle training, and standard rehabilitation therapy, in the experimental group, and conventional interventions such as simple joint movements or daily stretching, in the control group; and (4) outcome measures, including:

Global function: the ALS functional rating scale/revised (ALSFRS/ALSFRS-R).
Motor function: manual muscle strength testing (MMT), the UK medical research council (MRC) scale, maximum voluntary isometric contraction (MVIC), Ashworth spasticity scale (ASH), functional independent measure (FIM), checklist individual strength (CIS), the six-minute walk test (6 MWT), timed-up-and-go test (TUG), the two-minute walk test (2 MWT), the 5 repetition sit to stand test (5 STS), and grip strength test.
Respiratory function: arterial oxygen partial pressure (PaO_2_), arterial partial pressure of carbon dioxide (PaCO_2_), peak expiratory flow (PEF), maximal inspiratory pressure (MIP), maximal expiratory pressure (MEP), maximal voluntary ventilation (MVV), oxygen saturation on nocturnal oximetry (SpO_2_), forced vital capacity (FVC), maximum oxygen consumption (VO_2max_), forced vital capacity percentage predicted (FVC%), sniff nasal inspiratory pressure (SNIP), slow vital capacity (SVC), and electromyographic (EMG) signal of diaphragm/sternocleidomastoid muscle.
Sleep: Epworth daytime sleepiness scale (ESS).
Mental status: Hamilton rating scale for depression (HRSD) and Beck depression inventory (BDI).
QOL: EuroQol five dimensions questionnaire (EQ-5D), ALS specific quality of life-revised (ALSSQOL-R), McGill quality of life questionnaire (McGill QLQ), amyotrophic lateral sclerosis assessment questionnaire (ALSAQ-40), health survey short form (SF-36), impact on participation and autonomy questionnaire (IPA), 68-item sickness impact profile (SIP68), and Nottingham health profile (NHP).
Pain: visual analogue scale (VAS).
Swallowing function: the functional oral intake scale (FOIS) and eating assessment tool-10.
Fatigue: fatigue severity scale (FSS).
Family burden: caregiver burden scale (CBS).

Exclusion criteria included (1) non-RCT studies; (2) single case studies; (3) duplicated publications; (4) non-English publications; (5) studies without definite diagnostic criteria or incapable to extract valid data for analysis; and (6) low-quality studies.

### 2.3 Study selection and data extraction

Publications were independently selected by two researchers. The titles and abstracts were preliminarily screened, and then the full-text articles were read according to the inclusion and exclusion criteria for secondary screening. When disagreements occurred, a third researcher was consulted to reach an agreement. After the study selection, data were extracted independently by two researchers. Data, including first author, publication year, the country of study, the sample size, the basic characteristics of the subjects (such as age, course of disease, and severity of disease), interventions, observation time, and outcome measures, were extracted.

### 2.4 Assessment of quality

The quality of articles on RCTs was evaluated using the Jadad scale scoring system. Articles with a score of ≥3 were considered eligible for inclusion, while those with a score of <3 were considered low-quality (15) and not included. Eligible articles were classified and evaluated. Inter-rater reliability of the ROB tool has been demonstrated to range from fair to substantial depending on the assessment domain. The results of the risk of bias assessment will be summarized narratively with full assessments included in the appendix. Risk of bias between studies (publication bias) will be assessed and presented as funnel plots.

### 2.5 Statistical analysis

Review Manager 5.4.1 software was used for meta-analysis. Measurement data were presented as mean difference (MD), enumeration data were presented as odds ratio (OR), and effects size were presented as point estimates and their 95% confidence intervals (CIs). The degree of heterogeneity was determined using the *I*^2^ test, and the included studies were considered homogeneous if *I*^2^ < 50% or *p* **>** 0.1, and analyzed using a fixed effect model (the Mantel–Haenszel method), and heterogeneous if *I*^2^ **>** 50% or *p* ≤ 0.1, and analyzed using a random effect model (the DerSimonian–Laird method). Besides, we conducted one-way sensitivity analysis to assess the influence of every included RCT on the total WMD or RR for results with more than 2 included studies. A significance level of *α* = 0.05 was used in this meta-analysis.

## 3 Results

### 3.1 Study selection

**Figure 1:**
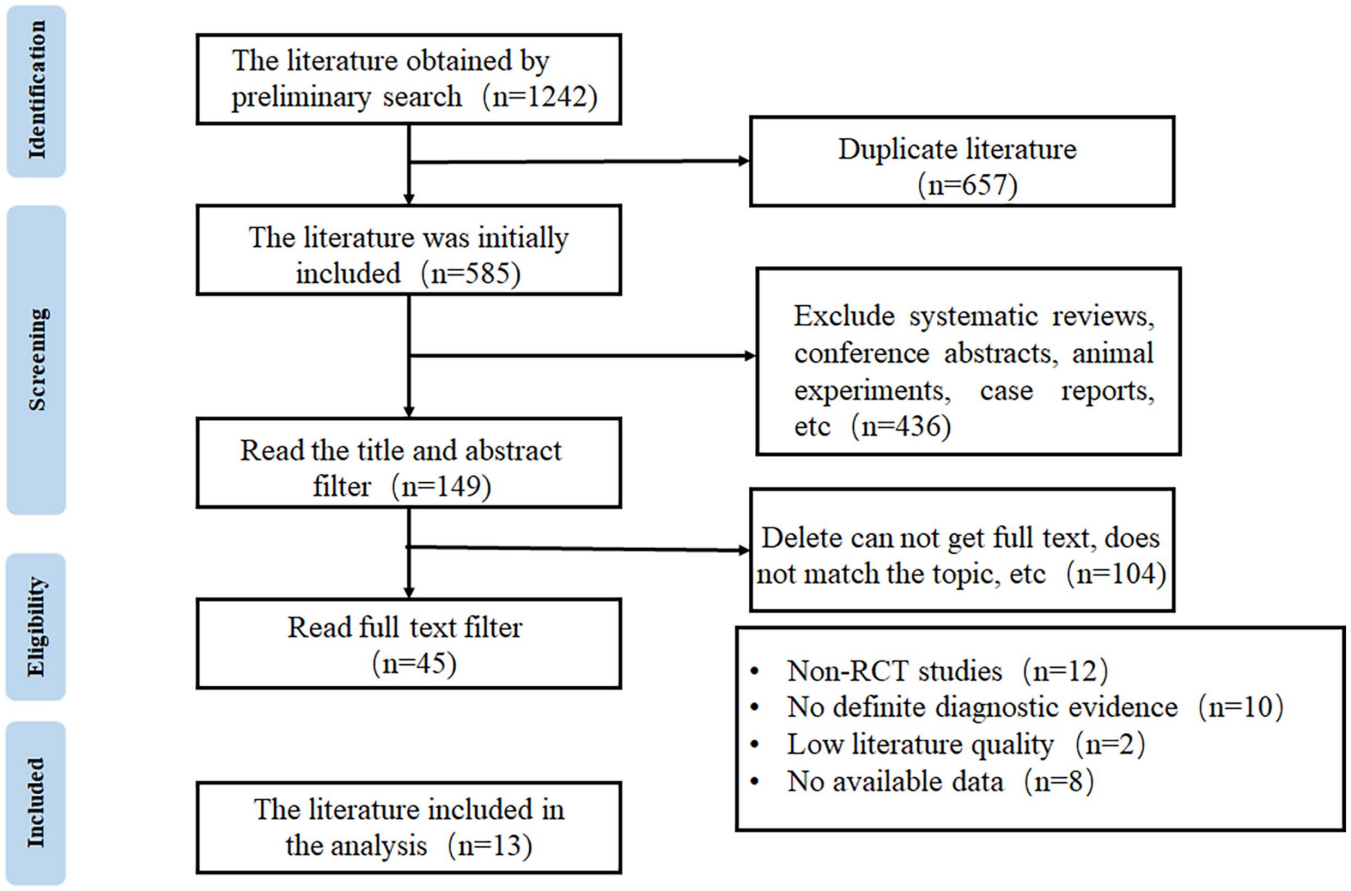
The flow chart of study selection.

A total of 1,242 articles were obtained through database search, from which 657 duplicated publications and 436 systematic reviews and case reports were excluded. Full texts were reviewed according to the eligibility criteria to exclude non-RCTs, studies without definite diagnostic criteria, and low-quality publications, and finally, 13 articles were included for meta-analysis (16–28). The flow chart of study selection is shown in Figure 1.

### 3.2 The basic characteristics and quality assessment results of the included studies

The 13 articles finally included were published between 2001 and 2021. A total of 657 patients with ALS were enrolled, with 299 in the experimental group and 358 in the control group. The intervention methods mainly included aerobic exercise (AE: exercise on a cycle ergometer, treadmill, or rowing machine under an intensity below the lactic acid threshold), resistance training (RT: active muscle exercise of endurance, strength, or power against gravity, external loaded weight, or fitness equipment), standard rehabilitation (SR: rehabilitation protocols that contained stretching, proprioceptive exercises, functional exercise, or any forms of sham (or placebo) treatments), passive exercise (PE: movement exercises induced by special therapists), expiratory muscle exercise (EE: expiratory muscle strength training or breathing exercise with the help of expiratory training devices) and daily activity (DA: maintaining usual daily activities) in the experimental group, and DA, SR and PE in the control group. Medium-intensity exercise training programs were developed based on parameters such as the target heart rate (THR) (i.e., the effective and safe exercise heart rate when aerobic exercise was used to improve the function of the cardiovascular circulatory system, and the range of THR was between 60 and 80% of the maximum HR), the Borg rate of perceived exertion (Borg RPE) (Exercise intensity was evaluated based on the degree of fatigue during exercise), and the repetition maximum (RM) (the maximum weight the individual can lift for a specific number of times), and guided training or home training was conducted by physical therapists.

Among the included studies, 4 were conducted in Italy, 2 in Portugal, 2 in the USA, 2 in Israel, and 1 each in Japan, Canada, and the Netherlands. The Jada scores of all RCTs included were 4–7, indicating that these RCTs were medium-high quality studies and met the inclusion criteria. The basic characteristics and quality assessment results of the included studies are shown in Table 1.

**Table 1:** Basic characteristics of inclusion in research and evaluation level of literature quality.

Include literature	Year of publication	Country	Sample size		Average age (year)		Disease course (month)		ALSFRS-R order of severity		Intervention measures		Completion method	Outcome indicators	Literature quality rating
			Experimental group	Control group	Experimental group	Control group	Experimental group	Control group	Experimental group	Control group	Experimental group	Control group			
Drory et al. (16)	2001	Israel	14	11	58.0	60.7	20.7	19.4	27.5	27.5	RT	DA	Experienced physical therapists evaluate patients and develop exercise plans, exercise at home, and provide weekly telephone follow-up and guidance	1, 4	4
Bello-Haas et al. (17)	2007	Canada	13	14	56.0	51.8	20.4	15.4	—	—	RT	SR	One physical therapist provides guidance and training at a rehabilitation facility	1, 3, 4	5
Pinto et al. (18)	2012	Portugal	13	13	57.1	56.8	11.5	12.6	34.4	33.5	EE	DA	Physical therapists provide weekly telephone guidance to patients and collect scale information	1, 5	7
Lunetta et al. (19)	2016	Italy	30	30	61.1	60.3	15.2	13.7	39.1	38.3	AE + RT	PE + SR	An experienced physical therapist guides patients to exercise at a rehabilitation facility	2, 3	6
Clawson et al. (20)	2018	USA	18	21	63.7	57.7	7.3	11.1	39.2	39.7	RT	PE	Physical therapists provide training to the patient’s family members and exercise at home, correcting and guiding them during each follow-up visit	2	7
Clawson et al. (20)	2018	USA	20	21	57.8	57.7	7.3	11.1	39.6	39.7	AE	PE			
Kitano et al. (21)	2018	Japan	21	84	62.8	62.7	26.4	18	41.1	38.1	SR + RT	DA	Training under the guidance of a physical therapist	2	6
Merico et al. (22)	2018	Italy	23	15	61.6	59.8	30.2	30.3	36.1	34.5	AE + RT	SR	Two experienced physical therapists guide patients to exercise in rehabilitation institutions	4	7
Braga et al. (23)	2018	Portugal	24	24	63.2	62.0	10.8	10.8	40.4	37.4	AE + SR	SR	The experimental group completed training under the guidance of a physical therapist in a rehabilitation institution; Control group trained at home	2	5
Zucchi et al. (24)	2019	Italy	32	33	65.14	64.74	15.7	16.6	39.8	40.2	AE + SR	SR	The entire exercise process at a nearby rehabilitation institution is supervised by a physical therapist. The experimental group has 5 sessions per week, while the control group has 2 sessions per week for a total of 10 weeks	2, 3	7
van Groenestijn et al. (25)	2019	Netherlands	27	30	60.9	59.9	15.5	18.0	42.3	42.3	AE + SR	SR	Home exercise/rehabilitation institutions guided by physical therapists	2, 3	6
Plowman et al. (26)	2019	USA	24	24	61.6	63.1	18.9	20.9	—	—	EE	DA	Guided by a physical therapist, patients are trained at home	2, 3, 5	6
Ferri et al. (27)	2019	Italy	8	8	50.7	55.5	20.5	13.4	40.4	35.0	AE + RT + SR	DA	The experimental group visited a gym under the supervision of two sports scientists, one doctor, and one medical student; The control group was required to maintain normal daily activities	2	4
Kalron et al. (28)	2021	Israel	14	14	58.5	60.4	7.3	6.4	35.7	37.5	AE + RT + SR	SR	Physical therapists and exercise rehabilitation personnel with over 10 years of work experience in the field of neurological rehabilitation in the treatment group are guided to practice in rehabilitation institutions; The control group practiced at home with the help of family members	2, 3, 4, 5	7

1 = ALSFRS (functional rating scale of ALS); 2 = ALSFRS-R (functional rating scale of ALS revised); 3 = FVC% (forced vital capacity percentage predicted); 4 = FSS (fatigue severity scale); 5 = MEP. AE, aerobic exercise; RT, resistance training; SR, standard rehabilitation; PE, passive exercise; EE, expiratory muscle exercise; DA, daily activity.

### 3.3 Meta-analysis results

After all the outcome measures reported in the studies included were summarized and the effectiveness and reliability of the meta-analysis were considered, outcome measures reported in at least 3 studies were selected and included in the meta-analysis. The outcome measures finally analyzed included ALSFRS, ALSFRS-R, FVC%, FSS, and MEP.

#### 3.3.1 ALSFRS

**Figure 2:**
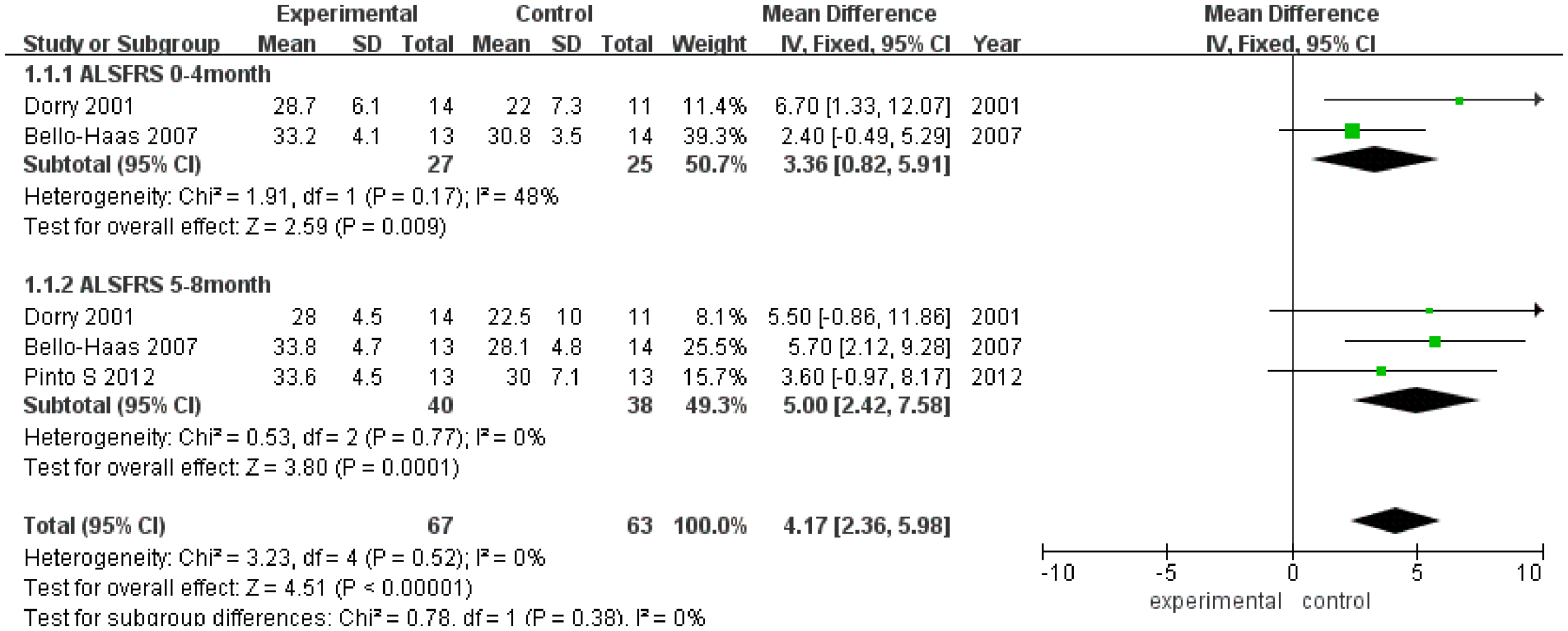
Forest plot of the effect of rehabilitation therapy on ALSFRS score in patients with ALS.

As shown in Figure 2, ALSFRS scores were reported in three studies, with no heterogeneity among these studies (*I*^2^ = 0%). Pooled analysis with a fixed effect model showed that the ALSFRS score of the experimental group was better than that of the control group (MD = 4.17, 95% CI: 2.36, 5.98, *Z* = 4.51, *p* < 0.001). Sensitivity analysis of ALSFRS score was carried out by eliminating single papers one by one. It was found that Pinto et al. (18) had a certain impact on the overall result, and the effect size increased (MD = 4.28, 95% CI: 2.30, 5.98, *Z* = 4.25, *p* < 0.001). Inter-group heterogeneity increased to 19.4% (Supplementary Figure S1).

Meta-analysis was further conducted in subgroups due to the differences in intervention cycles and follow-up duration. ALSFRS scores at 0–4 months of follow-up were reported in two studies (*I*^2^ = 48%), and the results showed that the ALSFRS score of the experimental group was better than that of the control group (MD = 3.36, 95% CI: 0.82, 5.91, *Z* = 2.59, *p* = 0.009). ALSFRS scores at 5–8 months of follow-up were reported in three studies (*I*^2^ = 0%), and the ALSFRS score of the experimental group was again better than that of the control group (MD = 5.00, 95% CI: −2.42, 7.58, *Z* = 3.80, *p* < 0.001).

#### 3.3.2 ALSFRS-R

**Figure 3:**
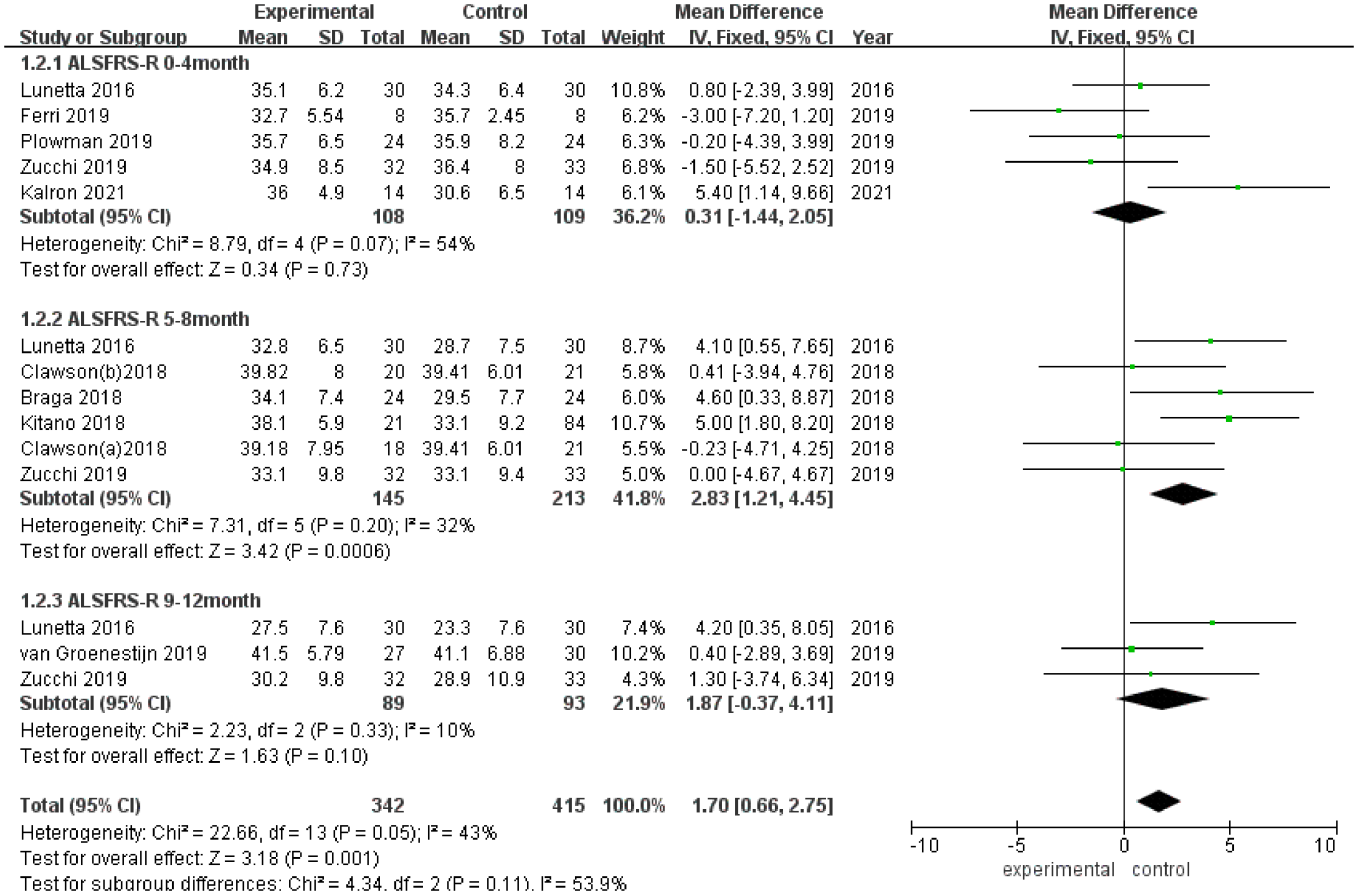
Forest plot of the effect of rehabilitation therapy on ALSFRS-R score in patients with ALS.

ALSFRS-R scores were reported in 8 studies (Figure 3), and there was a medium heterogeneity among these studies (*I*^2^ = 43%). A fixed effect model was used for pooled analysis, and the results showed that the ALSFRS-R score of the experimental group was better than that of the control group (MD = 1.70, 95% CI: 0.66, 2.75, *Z* = 3.18, *p* = 0.001). Sensitivity analysis of ALSFRS-R score was conducted by eliminating individual literatures one by one. It was found that Ferri et al. (27) had a certain impact on the overall result, with an increased effect size (MD = 2.02, 95% CI: 0.93, 3.10, *Z* = 3.65, *p* < 0.001). Inter-group heterogeneity decreased to 3.5% (Supplementary Figure S2).

Due to differences in intervention cycles and follow-up duration, subgroups were subjected to further meta-analysis. ALSFRS-R scores at 0–4 months of follow-up were reported in 5 studies (*I*^2^ = 54%), and no statistically significant difference in the ALSFRS-R score was observed between the experimental group and the control group (MD = 0.31, 95% CI: −1.44, 2.05, *Z* = 0.34, *p* = 0.73); ALSFRS score at 5–8 months of follow-up was reported in 5 studies (*I*^2^ = 32%), and the ALSFRS-R score of the experimental group was better than that of the control group (MD = 2.83, 95% CI: 1.21, 4.45, *Z* = 3.42, *p* < 0.001); and ALSFRS-R scores at 9–12 months of follow-up were reported in 3 studies (*I*^2^ = 10%), and the ALSFRS-R score of the experimental group was better than that of the control group (MD = 1.87, 95% CI: −0.37, 4.11, *Z* = 1.63, *p* = 0.10).

#### 3.3.3 FVC%

**Figure 4:**
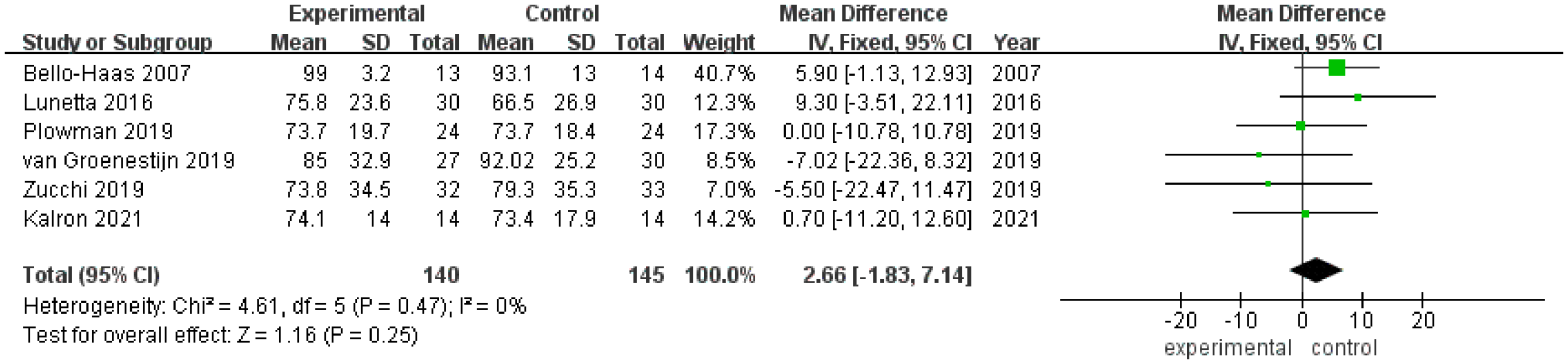
Forest plot of the effect of rehabilitation therapy on FVC% in patients with ALS.

FVC% values after rehabilitation therapy intervention were reported in a total of 6 studies, and no heterogeneity was noted among these studies (*I*^2^ = 0%). Pooled analysis was conducted using a fixed effect model, and the results showed no statistically significant difference in FVC% between the experimental group and the control group (MD = 2.66, 95% CI: −1.83, 7.14, *Z* = 1.16, *p* = 0.25) (Figure 4). Sensitivity analysis of FVC% value was conducted by eliminating single papers one by one. It was found that Plowman et al. (26) had a certain impact on the overall result, and the effect size increased (MD = 2.98, 95% CI: −1.86, 7.82, *Z* = 1.21, *p* = 0.23). Inter-group heterogeneity increased to 11% (Supplementary Figure S3).

#### 3.3.4 FSS

**Figure 5:**
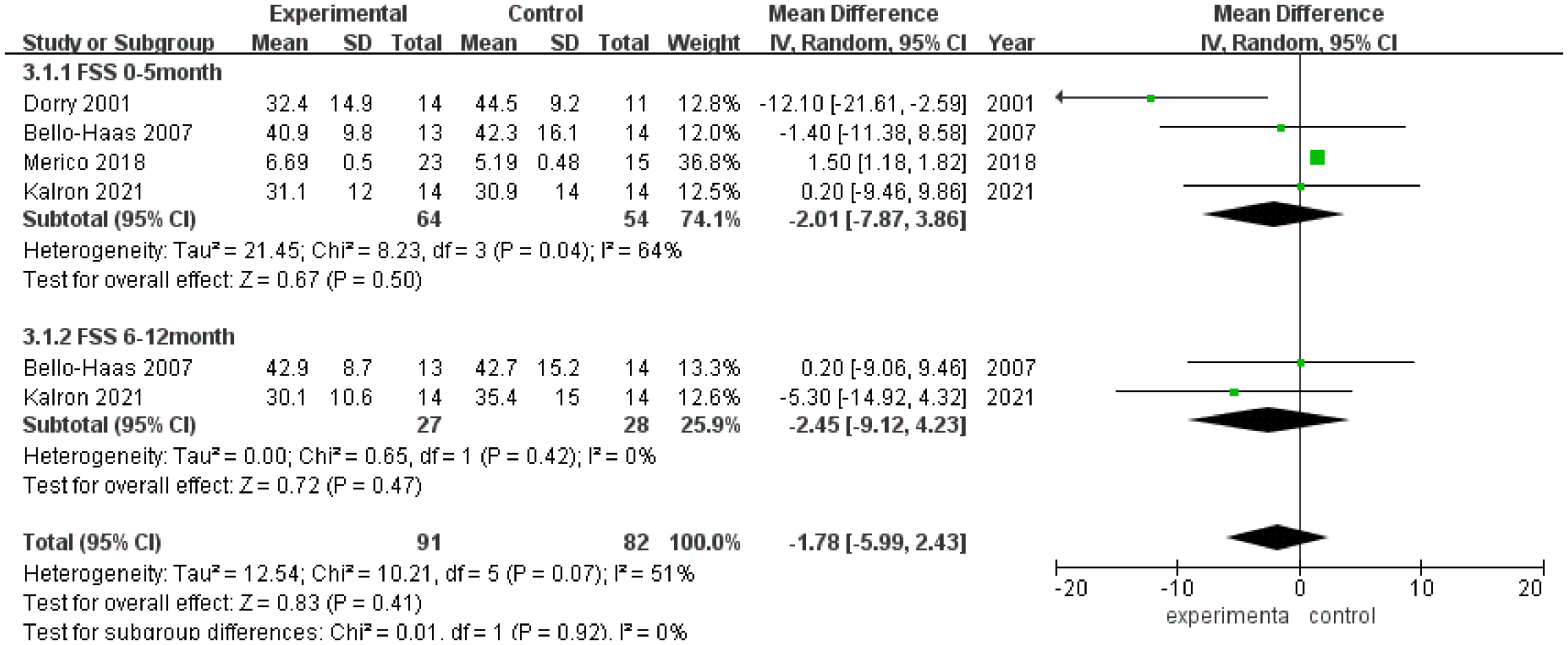
Forest plot of the effect of rehabilitation therapy on FSS in patients with ALS.

FSS scores after rehabilitation therapy intervention were reported in 4 studies (Figure 5), and significant heterogeneity was observed among these studies (*I*^2^ = 51%). Pooled analysis using a random effect model showed no statistically significant difference in the FSS score between the experimental group and the control group after intervention (MD = −1.78, 95% CI: −5.99, 2.43, *Z* = 0.83, *p* = 0.41). Sensitivity analysis of FSS score was conducted by eliminating single papers one by one, and it was found that Dorry et al. (16) had a greater impact on the overall result, and the effect size became positive, with statistical significance (MD = 1.49, 95% CI: 1.17, 1.80, *Z* = 9.20, *p* < 0.001). Inter-group heterogeneity increased to 25.2% (Supplementary Figure S4).

Meta-analysis was further performed in subgroups due to the differences in intervention cycles and follow-up duration. FSS scores at 0–5 months of follow-up were reported in 4 studies (*I*^2^ = 64%), and no statistically significant difference in the FSS score was noted between the experimental group and the control group (MD = −2.01, 95% CI: −7.87, 3.86, *Z* = 0.67, *p* = 0.50); and FSS scores at 6–12 months of follow-up were reported in 2 studies (*I*^2^ = 0%), and there was no significant difference in the FSS score between the experimental group and the control group (MD = −2.45, 95% CI: −9.12, 4.23, *Z* = 0.72, *p* = 0.47).

#### 3.3.5 MEP

**Figure 6:**
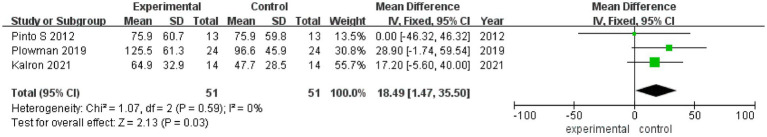
Forest plot of the effect of rehabilitation therapy on MEP in patients with ALS.

MEP values after rehabilitation therapy intervention were reported in 3 studies (Figure 6), with no heterogeneity among these studies (*I*^2^ = 0%). Pooled analysis was conducted using a fixed effect model, and it was found that the MEP value of the experimental group was significantly better than that of the control group after intervention (MD = 18.49, 95% CI: 1.47, 35.50, *Z* = 2.13, *p* = 0.03). The sensitivity analysis of MEP value was carried out by eliminating individual papers one by one, and it was found that the MEP result was relatively robust, and the removal of any study had no effect on MEP value.

#### 3.3.6 Publication bias

**Figure 7:**
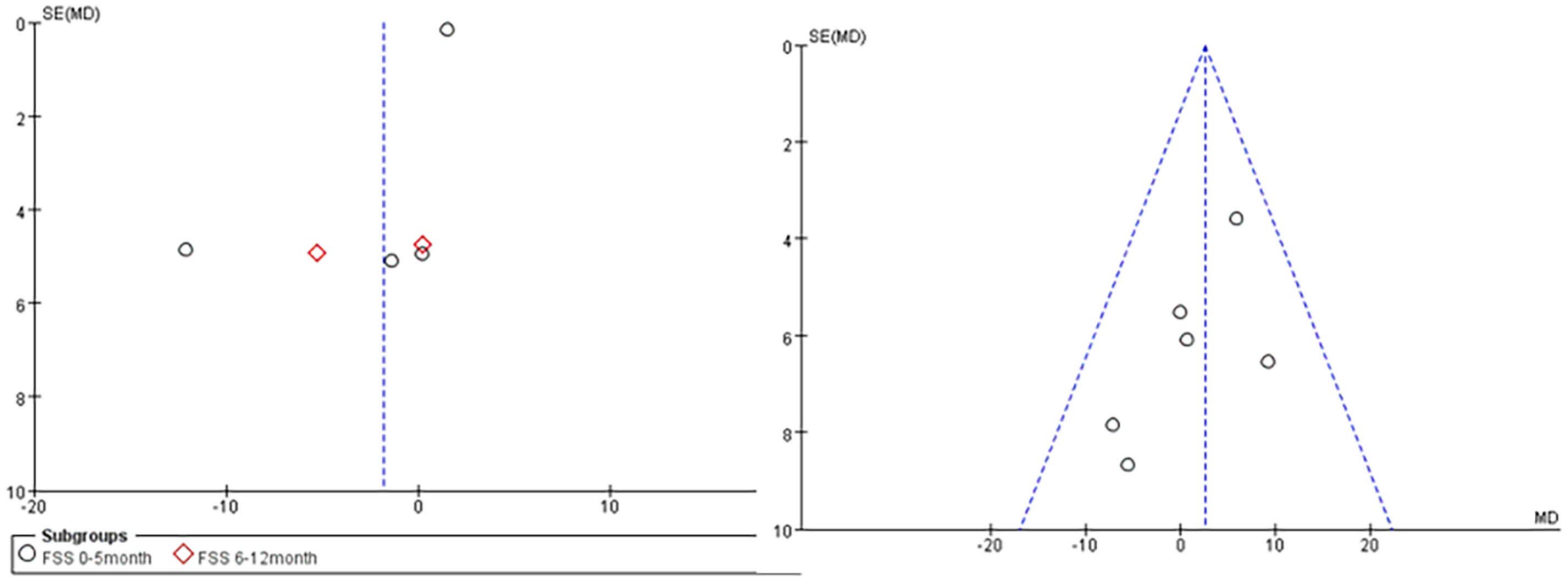
The funnel plots of FVC% (right) and the FSS score (left).

Publication bias was analyzed for 5 outcome measures (Figure 7), and the funnel plots of FVC% (right) and the FSS score (left) were significantly asymmetrical, suggesting the possible presence of publication bias.

## 4 Discussion

Systematic review and meta-analysis were used in the present study to investigate the effects of rehabilitation therapy on the global function, respiratory function, and QOL of patients with ALS to summarize these evaluation indicators. The results showed that the decreases in the short- (0–4 months) and medium-term (5–8 months) ALSFRS scores as well as the medium- (5–8 months) and long-term (9–12 months) ALSFRS-R scores were reduced and the MEF value was increased in the experimental group, in which rehabilitation therapies such as resistance training, aerobic exercise, respiratory muscle training were used, compared with those of the control group, in which conventional care was used, and no significant differences were observed in FVC% as well as FSS at 0–5 months and 6–12 months between the two groups.

The results of the present meta-analysis showed that rehabilitation therapy reduced the decreases of short-and medium-term ALSFRS scores as well as the medium-and long-term ALSFRS-R scores, suggesting that patients with ALS may benefit greatly from long-term rehabilitation training. Studies have found that regular physical exercise may increase muscle mass, induce astrocyte regeneration by activating these cells (a process that may change in ALS) (29), and slow down the decrease of the ALSFRS and ALSFRS-R scores. In addition, different effects of rehabilitation therapy on the ALSFRS and ALSFRS-R scores were observed in the same follow-up observation period. In the short-term follow-up (0–4 months), the decrease of the ALSFRS score in the experimental group was significantly slower than that in the control group, and no statistically significant difference in the ALSFRS-R score was observed. These differences were possibly explained by the evaluation of respiratory function, sitting breathing, respiratory dysfunction in the ALSFRS-R scale, and respiratory insufficiency, however, only occurred months to years after diagnosis of ALS (30). Therefore, respiratory function is not significantly affected in the early stage after rehabilitation intervention in patients with ALS. At the same time, the results of the present study showed that the MEP value was significantly increased after intervention in the experimental group compared with that of the control group (MD = 18.49, 95% CI: 1.47, 35.50, *Z* = 2.13, *p* = 0.03). Patients with ALS may experience respiratory dysfunctions such as dyspnea and cough weakness due to the progressive involvement of skeletal muscle and eventual respiratory muscle atrophy. Studies have shown that non-invasive ventilator treatment and death after pneumonectomy are the endpoints of patients with ALS (31). It can be seen that respiratory failure is the most direct and leading cause of death in patients with ALS. Therefore, the maintenance of respiratory function by various rehabilitation interventions and improvement of the symptoms of respiratory dysfunction are important parts of the rehabilitation treatment of ALS, and exercise training is the basis of pulmonary rehabilitation (32). Rehabilitation exercise training for the muscles of the whole body, muscles with residual strength, and respiratory muscles, such as aerobic training, endurance training, resistance training, and respirator training, were included in the present study. At the same time, indicators related to exercise intensity were used to determine the exercise program to avoid adverse reactions such as fatigue. Limb exercise can accelerate the delivery of oxygen throughout the body and maintain residual muscle strength, while respiratory muscle exercise can increase the activity as well as the strength and endurance of the diaphragm, and promote gas exchange, thereby improving the symptoms of dyspnea (33). In addition, the communication opportunities between doctors and patients are increased, and patients and their family members are encouraged and supported during the exercise training process, which improves the perception of social support in patients with ALS, and eventually helps to improve their QOL. Meanwhile, pieces of evidence suggest that exercise can mediate neural plasticity at the levels of dendritic reconstruction, axonal transport, synaptic transmission, neuromodulation, and nutritional factors (34), which may provide early protection for motor neurons in patients with ALS.

The severity of fatigue (the FSS score) was not increased in both the experimental group and the control group of patients with ALS in the early and middle stages, and no statistically significant difference in FVC% was noted between the two groups. Studies have shown that the structural and functional characteristics of respiratory muscle fibers change after training (35). Therefore, future multicenter and large-scale studies on the medium to long-term benefits of FVC% are needed. It was concluded in the present study that the application of rehabilitation therapy in the treatment of early to middle-stage ALS is feasible, with no exercise-related adverse reactions. The results of the present study were consistent with the findings of the study by Ferreira et al. (36), i.e., the global function and respiratory function of patients with ALS in the early and middle stages are improved after exercise training, with no adverse effects on disease progression and survival time.

ALS is a lethal rare disease. The number of RCTs retrieved in the present study was updated and increased on the basis of previous studies. However, the sample sizes of the included studies were still small, and defects that no placebo control can be set for exercise training existed. Furthermore, significant heterogeneities in the types, intensities, durations and outcome measurements of exercises may exist, and the medium-or long-term follow-up data were not provided in some studies. These factors may affect the final results of this meta-analysis. Due to the rapid progression of ALS and short survival time, patients will be in a vegetative state with only eye movement in the later stage without specific and effective treatments available, and the patients and their families are on the verge of giving up treatment. Therefore, the clinical benefits of early intervention with rehabilitation therapy in patients with ALS should be increasingly emphasized.

## Data availability statement

The original contributions presented in the study are included in the article/Supplementary material, further inquiries can be directed to the corresponding author.

## Author contributions

JC: Writing – original draft, Resources, Methodology, Funding acquisition, Formal analysis, Data curation, Conceptualization. XN: Writing – review & editing, Supervision, Resources, Methodology, Funding acquisition, Formal analysis, Data curation. HL: Writing – original draft, Software, Methodology, Investigation, Funding acquisition, Formal analysis, Data curation. QY: Writing – review & editing, Resources, Methodology, Investigation, Funding acquisition, Formal analysis, Data curation. KD: Writing – original draft, Software, Methodology, Investigation, Funding acquisition, Formal analysis, Data curation.

## Supplementary material

### SUPPLEMENTARY FIGURE S1

Sensitivity analysis of ALSFRS score.

### SUPPLEMENTARY FIGURE S2

Sensitivity analysis of ALSFRS-R score.

### SUPPLEMENTARY FIGURE S3

Sensitivity analysis of FVC% value.

### SUPPLEMENTARY FIGURE S4

Sensitivity analysis of FSS score.
